# Application of the paediatric medical traumatic stress model to the mental health experience of young people living with type 1 diabetes: a qualitative study

**DOI:** 10.1186/s12888-025-07760-w

**Published:** 2026-01-29

**Authors:** Rigel Paciente, Sara Coombs, Karen Lombardi, Helen Milroy, Keely Bebbington, Heather Roby, Craig E. Taplin, Nicole Wickens, Stella Rose, Alix Woolard

**Affiliations:** 1 The Kids Research Institute Australia, Perth, Australia; 2 The University of Western Australia, Perth, Australia; 3 Edith Cowan University, Perth, Australia; 4 Perth Children’s Hospital, Endocrinology and Diabetes, Perth, Australia

**Keywords:** Type 1 diabetes, T1D, Pediatric medical traumatic stress, PMTS

## Abstract

Despite the various traumatic events that a young person living with type 1 diabetes (T1D) may experience, little is known about the burden and manifestation of traumatic stress in this population. Though mental health outcomes have been explored generally, medical trauma-sensitive approaches to understanding these experiences remain limited. We utilised a qualitative descriptive approach to explore the impact of T1D on young people’s mental health through the paediatric medical traumatic stress model (PMTS). Young people aged 13–30 living with T1D were interviewed. These interviews were recorded, transcribed, and analysed using Reflexive Thematic Analysis. Three main themes were generated: (1) perceived sources of trauma throughout the T1D journey, (2) responses to and consequences of trauma, and (3) role of resilience throughout the T1D journey. The first and second themes elucidate the sources of traumatic stress ranging from physiological, clinical, and social experiences. Furthermore, the consequences of unaddressed trauma are observed throughout adolescence, but is most evident in the transition between adolescence and young adulthood. The final theme refers to sources of resilience for young people living with T1D and its role in mitigating the consequences of adverse trauma immediately post-diagnosis and in the long term. Application of the PMTS model to these experiences offers a novel perspective on young people’s experiences of living with T1D and enhances our understanding of trauma and traumatic stress in this population. Findings suggest that interventions focused on bolstering resilience might contribute to the prevention of long-term adverse mental health outcomes for young people living with T1D.

**Supplementary Information:**

The online version contains supplementary material available at 10.1186/s12888-025-07760-w.

## Clinical trial number

Not applicable.

## Introduction

Paediatric medical traumatic stress (PMTS) refers in part to the psychological and physiological responses of young people to illness, injury, and medical procedures [1]. These psychological responses can be referred to as manifestations of post-traumatic stress symptoms (PTSS) which are grouped into arousal, negative thoughts or moods, avoidance, or re-experiencing of events. Not all symptom groups are required to be present, which differentiates PMTS from a full diagnosis of post-traumatic stress disorder (PTSD; requiring the presence of symptoms from each group for a diagnosis). Common experiences of PMTS often culminate in avoidance or complete withdrawal of much needed care due to maladaptive coping responses adopted throughout development, especially for those living with chronic conditions. The PMTS framework also describes the effects through developmental stages, such that unimpeded progression through the PMTS model can further deteriorate PTSS at older ages if the young person continues to adapt using unhelpful coping strategies. Conversely, recovery from PMTS (referred to as ‘post-traumatic growth’ [PTG]) can also be encouraged through more positive mental processes and coping [1].

The diagnosis and ongoing management of type 1 diabetes (T1D) might contribute to the development of PMTS. The psychological sequalae of living with T1D are well documented including anxiety, depression, diabetes burnout (i.e., emotional exhaustion and disconnect due to living with diabetes), and psychological distress [2, 3]. The ongoing demands of living with T1D including vigilance around food consumption, insulin intake, risks of hypoglycaemia, effects of exercise, potentially painful medical procedures and frequent hospital visits are often cited as a cause of these mental health concerns. Additionally, life stressors during adolescence and early adulthood are compounded for those with T1D [4]. The presence of significant psychological distress is known to negatively impact diabetes management and physiological health outcomes [5, 6].

To date, the experience of trauma and traumatic stress responses associated with T1D have not been adequately explored. Some studies have attempted to identify PTSS generally among people living with T1D. For instance, Hosoda-Urban and O’Donnell found that 28% of their clinical population experienced clinically significant PTSS that had been attributed to a diabetes-related event (e.g., hypoglycaemia, diabetic ketoacidosis [DKA], or hospital visits), and that the number of visits to the emergency room due to a diabetes-related event moderates this relationship [7]. In another study, PTSS was also common among children living with T1D, with a high frequency of hypoglycaemia noted as traumatic using the Child Posttraumatic Stress Reaction Index [8]. Another study found similar rates of PTSS, noting that 31% of their participants experienced PTSS that was directly related to episodes of hyperglycaemia [9]. The prevalence of PTSS could be higher granted the limitations of existing PTSS questionnaires which inaccurately capture the entire T1D experience [7, 9]. Exacerbating this issue is the lack of consensus regarding the use of diabetes-specific patient-reported outcome measures tracking mental health outcomes, most of which do not specifically address PTSS [10].

Unresolved PMTS is a significant risk factor for the later diagnosis of PTSD, particularly for those who are regularly exposed to invasive medical procedures that might trigger trauma responses, as is the case for young people living with T1D [1]. Furthermore, there is evidence that diagnosis with PTSD is associated with poorer diabetes-related outcomes, including higher HbA1c (glycated haemoglobin), increased risk of DKA, and more frequent and longer hospital stays [11]. Given the serious negative health consequences of PTSD or PMTS for young people living with T1D, it is imperative that early symptoms can be detected, and appropriate early interventions offered. Currently, we lack the thorough understanding of the experience of PMTS in young people with T1D, necessary to guide these efforts. Hence, this study aimed to understand, within the context of PMTS, the mental health experiences of young people living with T1D. To do this, we asked the following research questions:

1. What are the characteristics and experiences of PMTS in young people living with T1D?
2. What factors are associated with increased risk of progression of PMTS or impaired post-traumatic growth (PTG)?
3. What are the behaviours, psychological processes and constructs explained by young people living with T1D that might help mitigate the development of PMTS?

## Methods

This study received ethical approval from the Child and Adolescent Health Service Ethics and Governance committee (RGS0000006378). Informed consent was obtained from all participants. Consent was acquired from both the young person and their parents if they were less than 18 years old and acquired only from the young person if they were 18 years old or above, and confirmation of consent immediately prior to data collection. A qualitative descriptive approach was used to investigate the aim and subsequent research questions. The theoretical lens of PMTS was used throughout the investigation, as described by Kazak et al. [1].

The study used convenience and snowball sampling for recruitment to account for the relatively small population of young people aged 13 to 30 years old living with T1D in Western Australia. The primary form of recruitment was through a member of the research team approaching young people during their clinic appointments in the Perth Children’s Hospital. The research team also utilised the hospital’s research and clinical database to recruit young people living with T1D via email and phone calls where previous consent to be contacted for research opportunities had been provided. Social media was utilised, with allied T1D organisations reposting appropriate recruitment materials on their social media pages. Finally, a snowball form of recruitment eventuated through existing participants referring other young people living with T1D in their personal networks.

Eligible participants were young people living with T1D in Western Australia, aged between 13 and 30 years of age, with a diabetes duration of > 1 year at time of enrolment. Young people with T1D and concurrent mental health concerns were not excluded. The age range of 13 to 30 years was included to capture a broad range of experiences throughout adolescence, young adulthood, and the transition between the two. In addition, the following reasons were used to justify the broad age range: (1) the inclusion of only adolescents might not capture that transitional experience, and (2) the inclusion of only young adulthood may not be reflective of current experiences of medical settings and might be more prone to recall bias.

Semi-structured interviews were conducted to ascertain mental health experiences related to living with T1D, with particular emphasis on potential sources of trauma and psychological sequalae. All interviews took place over Microsoft Teams [12], and were recorded and transcribed verbatim. Interviews ranged from 40 to 70 min. In addition, the researchers (RP, SC) involved in interviewing participants recorded field notes throughout each interview. Interview questions explored participants’ experiences at time of T1D diagnosis and ongoing management of T1D. Key concepts explored included, but were not limited to, the emotional and behavioural response to diagnosis; regular emotional and behavioural responses to management of T1D; how T1D impacts daily life; and perceptions of the future with regards to living with T1D. The interview guide is included as a supplementary file. All data collected (audio recordings and transcribed materials) were saved in secure servers accessible only by the research team. Hospital admission at and post-diagnosis and diagnostic experiences of the participants were collected to better contextualise the participants’ experiences with T1D which might contribute to long-term trauma in participants [7–9].

The transcribed interviews were analysed using Braun and Clarke’s Reflexive Thematic Analysis [13]. Analysis was guided by the theoretical framework and assumptions underlying PMTS particularly during theme and subtheme refinement. After each interview, RP collated all field notes taken and began analysing initial concepts which were discussed. Interviews were transcribed and uploaded to NVivo which was used to support the analysis. RP familiarised themself with the transcripts and generated initial codes across segments of texts that summarised participant experiences. These codes were refined and (where appropriate) merged based on similarity and interpretation of the concepts until initial subthemes and themes were formed. Each theme and subtheme were refined and named as more participant interviews was analysed until no changes to the theme and subthemes could be made.

To ensure rigour, Guba and Lincoln’s methods were applied throughout the entire analytical process [14]. Memos were kept by RP throughout the interview and analysis processes to track thought processes and ensure reflexive practices. This aided in the sense making of participant experiences with respect to the PMTS framework, RP’s pre-conceived ideas of T1D diagnosis and management and potential influence in analysis, code naming and theme generation. These memos would then aid in triangulation as these were written thought-processes contributing to analysis. Sense making of the data, the coding process, and preliminary theme/subtheme generation were triangulated between RP and the research team to ensure that the formation of themes was logical (i.e., logic of coding and how codes related to make preliminary themes/subthemes was coherent) and reflected the experiences of the participants without excessive abstraction. Finally, themes and subthemes were presented back to the participants (i.e., member checking) to ensure that results reflected their experiences. Themes were also reviewed by the research team prior to write-up and submission. The Standards for Reporting Qualitative Research were adhered to throughout manuscript writing [15].

For transparency in relation to the researcher perspective, the lead author of this manuscript (RP) does not live with T1D but has close relatives who live with both T1D and type 2 diabetes. They are also engaged in other research in T1D and mental health which may influence their interpretation of the data in the present study. This perspective has been explicitly acknowledged throughout the analysis by ensuring that a reflexive approach is adopted and memos documenting this were kept throughout. Finally, the lead author is an experienced qualitative researcher with prior experience in Reflexive Thematic Analysis.

## Results

Eleven young people took part in the study. The characteristics of participants are shown in Table 1.

**Table 1:** Summary of participant characteristics

Characteristics	*N*(% or SD)
Total number of participants	11
• Adolescents (‘A’, 13-17yrs)	4 (36.4)
• Young Adults (‘YA’, 18-30yrs)	7 (63.6)
Age (Mean ± SD)	19.2 ± 4.1
Gender (F)	7 (63.6)
Diabetes Duration in yrs (Mean ± SD)	7.45 ± 6.4
Experience of DKA at Dx	3(27.3)
Admission at Dx	8(72.8)
• Emergency Department	3(27.3)
• Intensive Care Unit	3(27.3)
• Other	2(18.2)
Admissions since Dx	2(18.2)

Note. Dx = diagnosis. DKA = Diabetic Ketoacidoses. ‘Other’ under Admission at Dx denotes that the clinical database did not have data on reason for admission

**Fig. 1:**
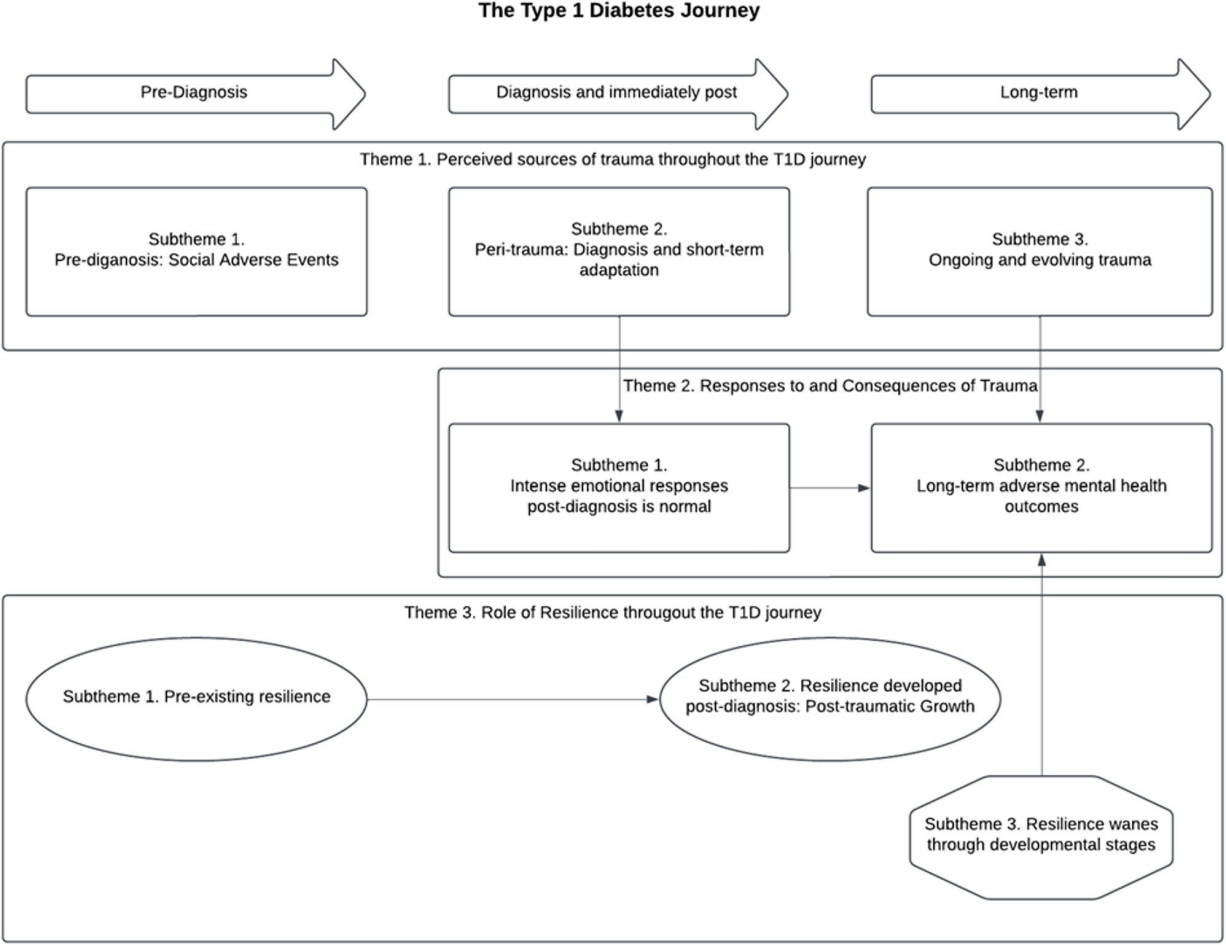
Thematic map showing different stages of the T1D journey and the relationship between the themes. *Note.* The arrows depict how the young people have spoken about the relationship between each theme

Three main themes were generated from the data congruent with the PMTS model: (1) sources of trauma along the T1D journey from pre-diagnosis through to long term management, (2) adaptive and maladaptive responses to trauma across the T1D journey and consequences for mental health in the long term, and (3) the role and sources of resilience to overcome the challenges of living with T1D. A summary of the themes and subthemes is presented in Table 2. Consistent with assumptions of the PMTS model, the social aspects of the journey were also perceived as sources of trauma [1]. The relationship between these themes and subthemes are also displayed in Fig. 1.

**Table 2:** Summary of themes and subthemes

Theme	Subthemes
Perceived sources of trauma throughout the T1D Journey	• Pre-diagnosis: Social adverse events • Peri-trauma: Diagnosis and short-term adaptation • Ongoing and evolving trauma
Responses to and Consequences of Trauma	• Intense emotional responses post-diagnosis is normal • Long-term adverse mental health outcomes
Role of resilience throughout the T1D journey	• Pre-existing resilience • Resilience developed post-diagnosis: Post-traumatic growth • Resilience wanes through developmental stages

### Theme 1. Perceived sources of trauma throughout the T1D journey

This theme encompasses the perceived sources of trauma along the T1D journey. The journey is defined as commencing pre-diagnosis and extending through diagnosis to long-term management. The sources of trauma and associated psychological sequalae are not only tied to the physiology and physical ill health that is caused by T1D, but from social circumstances as well.

#### Pre-diagnosis: social adverse events

Most young people described a substantial period of time before their diagnosis where the symptoms of T1D began. The severity of the symptoms varied. Some participants whose symptoms were more severe (e.g., vomiting, very frequent urination, lethargy) noted that they were met with reprimand and a lack of compassion from their social systems. These systems included parents, teachers, and other authority figures, for example, in their extracurricular activities, due to a lack of understanding of diabetes-related symptoms at the onset. This adverse social interaction with support systems was perceived to be a source of trauma.

*One night*,* I vomited… we had these three doors*,* bathroom*,* laundry and toilet*,* and they were all close and I was rushing to get to the toilet and I just absolutely vomited over all three doors… [mum] says that this is her deepest regret but she was so mad at me that she made me sit there and clean it… I was like 10 and then I was just still throwing up so much. (YA1)*

For a handful of participants, this initial adverse response from their support systems influenced their outlook and perspective of living with T1D well beyond diagnosis. This included navigating their relationships with the same people with regards to their involvement in the management of T1D and transfer of care between the parent and the young person. This was common among young people who were cognisant of their pre-diagnostic experiences.

*Even to this day*,* sometimes still just love her to bits*,* but there are moments where… I don’t need to be micromanaged… I’m fully capable of doing this myself*,* like it’s been 19 years this year… I would like some aspect of you registering that and being able to trust that I can keep myself alive… she felt guilty for so long. (YA4)*

#### Peri-trauma: diagnosis and short-term adaptation

Most of the young people who perceived their diagnostic experience as traumatic identified interactions with the healthcare team as a contributing factor. Young people who were cognisant of their diagnostic experience reported that information about their condition and diagnosis was withheld from them and their parents, at times, even when they directly sought clarity from the clinical team.

*First we went to the GP… they finger pricked me and they didn’t tell me what was going on. They just said to go to ED and then we spent a week in hospital. I was really scared [in ED] because they didn’t actually tell me. (A1)*

Surprisingly, only one person identified their symptoms at the time of diagnosis as a source of trauma.

*I do remember saying to myself because of how sick I was. I was like in my head going*,* ‘God kill me now*,*’ like I would literally rather die… I was getting up every 20 minutes to*,* like*,* violently throw up. (YA1)*.

#### Ongoing and evolving trauma

Young people stated that T1D management was a recurring source of trauma, due to both the invasive medical procedures associated with medical management (e.g. administration of insulin, site changes) and their interactions with the clinical care team. With regards to medical management, young people noted experiencing anxiety related to needles and the use of medical devices.

*I sat there and forgot I was connected and had the old Medtronic pumps with the files*,* and I was sticking a pen up the end*,* pushing the insulin it*,* forgetting it was still connected to me just because I was like*,* ‘oh*,* look at that.’ I was then really low overnight. Dad was next to me*,* waking up every 10 minutes*,* feeding me lemonade and yogurt and toast and this and that… Then uh*,* line failed a couple of months later*,* later in the year. I ended up having one of the Glucagon pens and mom ended up having to call ambulance and everything. (YA4)*

*I think that’s one of the things that worried me… ‘cause they went through the insulin pen and stuff and they started talking about pumps*,*but they used the word cannula… and at that point I was in the hospital with one of the blood cannulas in my arm. So I pictured that being permanent*,*so that kind of freaked me out a bit (A2)*

Young people also described the lasting impact of the clinical practices required at the time of diagnosis. Young people realised that clinicians are simply acting upon what is currently best practice when managing T1D, but that these were inherently traumatic and caused hypervigilance.

*Yeah I was pinned down on the bed… I remember the doctors holding me down…Certain doctors now I struggle to see because I’m like you know*,* took me a while to figure out and it’s going to sound awful but [the doctor] was of a certain race and any doctor of that race I was like*,* ‘you can’t*,* you’re going to hurt me*,*’ it sounds so awful saying it. (YA4)*^1^

Young people described stigmatising experiences in relation to their T1D. Young people noted that the lack of knowledge within the general population about T1D (causes and management) contributed to adverse social interactions. Young people were also cognisant of their differences to their healthy peers, which fuelled resentment towards T1D and others who were assisting with their care.

*I had some… I think it was to do with sugar control had some sores there [in mouth]. I think it was acne or pimples on and they’re [classmates] like*,* “getaway type one cancer*,*” or something like that. (A1)*

Finally, a small number of participants stated that the necessary vigilance around diet contributed to anxiety. This was true particularly for those that were also living with coeliac disease (i.e., adverse reactions to gluten).

*I remember my dad made dinner*,* and you know it was really great… but I remember having to yell at him being like “I can’t eat this*,* there’s carbs in that*,* this could kill me – I’m going to die. (YA6)*

*Food choices are extra hard because everything that’s gluten free is really high in carbs so I’ve struggle with my levels… I’m always getting spikes no matter what I do which is really annoying and hard… I can’t get low carb items because they all have gluten in them… I’m always needing more insulin and just because I’m coeliac as well. (YA5)*

### Theme 2. Responses to and consequences of trauma

This theme describes the young people’s psychological responses to the perceived sources of trauma experienced throughout their T1D journey as detailed in Theme 1. Importantly, consistent with the PMTS model, they described both adaptive responses that appeared to be part of adjusting to the diagnosis and ongoing management of T1D, and maladaptive responses that appeared to be linked with negative physical or mental health consequences.

#### Intense emotional responses post-diagnosis is normal

Young people described the emotional response to diagnosis and ongoing management which were perceived as normative and indeed necessary responses to living with T1D. The initial adjustment period after diagnosis brought with it feelings of grief and loss of the life they thought they were going to live.

*I guess it’s almost like a lost feeling*,* like*,* I’ve lost the life I had before… it will probably change as*,* you know*,* I am further into my diagnosis and it becomes more normal. But I kind of always feel sad about it because at this point*,* it’s literally till the day I die… I guess I feel really sad that [T1D] is forever. (YA5)*

As such, part of the adaptation process for young people was coming to terms with living with T1D for the rest of their lives. However, the notion of ‘acceptance’ as part of coping with T1D was queried. Engagement with daily diabetes management tasks was seen as a necessity, rather than an active choice related to acceptance of their diagnosis. Given this perceived lack of choice, it was debated whether ‘acceptance’ was the right word to define coping with T1D.

*[Acceptance] is… you’re not doing these things because you want to. It’s this or I die… I don’t have an option whether I do this or not and that’s what I mean in terms of the acceptance thing*,* it’s not really you accept. (Y4)*

Since adapting to the needs of T1D was not necessarily an act of ‘acceptance’, this severely impacted the way that the participants related to T1D and their view of themselves in relation to T1D. In turn, this impacted their willingness to engage with their T1D management, how they navigated their psychological needs, as well as social interactions that involves discussing their T1D.

*In the hospital… I was like*,* ‘nope*,* not doing this*,*” and then when I got home*,* I was like*,* ‘oh*,* I have to do this’… I just… I kinda got into a routine and didn’t really concentrate… I guess I had to do it because I didn’t really have a choice. (YA2)*

However, young people also described instances of maladaptive coping, including intentional mismanagement of their diabetes as a behavioural response for distress. For instance, purposely over-administering insulin in response to the distress caused by persistent high blood glucose levels, known as “rage bolusing”. Over-administration of insulin can lead to potentially serious hypoglycaemia.

*So previously what I did was instead of psychologically being OK with it then I did something physically. So then I did something called*,* I don’t know if you know this term*,* but I did rage bolus. So give a bunch of insulin to get down*,* so physically doing stuff or if I’m low and I’m really angry that I’m low*,* I just like eat as much as I can or something. (A4)*

#### Long-term adverse mental health outcomes

Young people described poor mental health outcomes associated with the stress that comes with living with T1D. These outcomes manifested responses that were maladaptive and particularly detrimental to their diabetes management. The young people noted experiences that closely mirrored post-traumatic stress symptoms. For example, young people referenced experiences most like internalisation symptoms. These symptoms included hypervigilance, hopelessness, and how they perceived that they were a burden to others. For perceived burdensomeness, this made it more difficult to be willing to receive help from others.

*I kind of get really worried and like sometimes I even pull over to check my levels… if I’m doing an hour drive*,* I’ll end up stopping like four times. Which is so time consuming but I just I have to because otherwise I get too anxious about it and yeah*,* so I guess it adds to that a lot. (YA5)*

Avoidance was also referenced by a few of the young people, particularly surrounding engagement with their clinical care team and in self-management of T1D.

*[Avoidance] was all within those teen years… I got reprimanded quite a lot at the clinics and scolded… I didn’t want to do anything that will impose on my vanity. So I was like*,* “oh well*,* I’m just gonna pretend I don’t have [T1D]. So I just stopped blousing for my lunch because I didn’t want boys to think I was different… So everytime it would come to getting my HbA1c*,* I would be like*,* ‘no*,* I don’t wanna do it’… Soi I really did not like going to clinics because then it was gound out that I wasn’t adequately taking care of myself. And also again*,* leading into the whole medical trauma side*,* I was never met with*,* ‘I understand that this is difficult for you’… so I did resent it a lot because I was like… they don’t understand. (YA1)*.

Some people noted depression, self-harm, and suicidal thoughts and behaviours as an extreme psychological sequelae for the unaddressed medical trauma. A small number of participants refer to feelings of hopelessness and stress which are known determinants to suicidal ideation.

*I’ve had a history of anxiety and depression as well as suicidal ideation… all of these are contributed to by the effects of living with T1D and the stress it adds to the daily routine. (YA4)*.

*[Disclosing to their psychologist] This is great*,* one day*,* I’ll be dead but I won’t have Type 1. (YA7)*

*Yes I would say that [T1D] is a contributing factor… I have been diagnosed with depression… I don’t think that diabetes is not necessarily the cause but it does hinder being able to get better because it’s just another layer of like*,* well*,* this feels hopeless. (YA1)*

### Theme 3. Role of resilience throughout the T1D journey

This theme describes the sources and the role of resilience to help cope with the demands of T1D. In particular, the acknowledgement that young people and their psychological processing post-diagnosis is heavily influenced by the pre-existing psychological processing, which in turn was likely influenced by their support systems (i.e., family and close friends).

#### Pre-existing resilience

Young people identified that the resilience built prior to diagnosis was heavily influenced by their immediate family and this resilience carried them through the initial stages of adaptation to T1D. This resilience also persisted well into the later stages of management. Related to the concept of “acceptance”, some young people were swift to acknowledge that they must engage with their T1D management rather than wallowing in feelings of despair. Some young people noted that because of their pre-existing resilience, even at the point of diagnosis, their outlook on their life with regards to T1D was not impacted.

*I never remember feeling any type of resentment towards the diagnosis. I kind of took it in stride… this isn’t going to hold me back… I remember feeling at the time like*,* “oh hell yeah*,*” like I got this like… that’s fine. (A1)*

#### Resilience developed post-diagnosis: Post-traumatic growth

Post-diagnosis, young people reported that the ongoing practice in self-management and routine allowed them to feel a sense of independence and control over the management of their T1D. This sense of independence was a form of resilience as in their view, it normalised T1D and allowed them to develop a better understanding of their future.

*I think it’s just a bit of a routine thing. I think it definitely changes your day-to-day life. But as you get used to it*,* it just becomes… just normal things you have to do. (A1)*

Young people noted the importance of peer support from those living with T1D and those living without. For peers living with T1D, it was perceived to be beneficial as it normalised T1D for them and provided someone they can relate to and share information with. Information shared was related to management of the T1D directly or skills to help navigate the emotions of living with T1D. Peers living with T1D were found either incidentally through school, extracurricular activities and T1D-specific community centres.

*There’s a lot of camps going on that [hospital] you might have some great people in the same situation as you. You know also having a bit of trouble with mental health or trauma and stuff*,* but also having to deal with the diabetes*,* you know*,* or trauma from diabetes… For them*,* it would probably be nice to have the same people in the same boat. You know*,* like they say*,* can vent to or*,* you know*,* talk to and stuff. (YA1)*

Peers and support people without T1D were also supportive in both the practical and emotional sense, which surprised participants. The young people’s peers and support people without T1D became quickly accustomed to the T1D routine. Parents played a central role in supporting young people developing resilience, particularly when it came to helping manage and scaffold the day-to-day of living with T1D.

*So yeah*,* [mother] was doing the site changes*,* it was only a month ago when I could do the site changes by myself so she always did them and so yeah… So at the start my dad was always the one that gave the Lantus [medication] ‘cause at the start I was on injections. Yeah*,* so I did my normal injections from the start*,* but my dad did the Lantus. My dad did the CGM. My mum carb counted everything*,* so I think maybe in year 8 [aged 12–13 years]*,* that’s when I started managing it more by myself and progressively it just got better and better. I think in year 10 [aged 15–16 years] I could do my own CGM and then later on this year I could do my own site changes… It was really good. It was*,* it was so*,* so gradual. Yeah*,* I think that that helped so much. It was mainly my mum that was taking it all. (A4)*

Young people also noted the change in mindset which allowed them to practice resilience in ongoing management of their T1D. These changes manifested in different ways. For some, it was a shift in their own perception of their capabilities with regards to managing T1D. In this way, they were also able to improve their relationship with T1D while still being able to acknowledge that management can be difficult – albeit they were adequately equipped to respond to these challenges.

*Definitely a lot of times like I was kind of fed up with it and just was like… a bit overwhelmed about having to do this for the rest of my life but then obviously I’ve come to the realisation I’ve done this for my whole life already. I’m pretty sure I can do this going into the future as well. (YA2)*

#### Resilience wanes through developmental stages

While all young people described how they were able to develop resilience throughout their adolescence, the young adults identified that their resilience waned during the transition between adolescence and young adulthood. Primarily, this was due to the complete transfer of care from parents to the young person, on top of the new responsibilities that one naturally acquires at young adulthood.

*But again*,* I don’t know why this would be*,* but I think that I really would have struggled a lot more and had so much resentment and everything if I was diagnosed at an older age. I don’t know why*,* if that’s just because of life and I’ve become in all aspects of my life I’ve become less resilient*,* especially in regards to mental health. Like something will happen and it will throw me over the edge. (YA1)*

In addition, the young adults noticed a resurgence of inadequately addressed self-stigma as they became more cognisant of their differences compared to their peers. Self-stigma in the context of chronic diseases can be conceptualised as a negative perception of self that is inherent due to their chronic condition regardless of whether this is true (e.g., devaluation and low expectations of self especially when comparing themselves to others without chronic conditions; [16].

*Yeah, I guess sometimes I feel sick. I know it's not really the right word to say, but I guess I just feel like yeah, like that like I'm a sick person and that I always need my insulin which essentially is medicine right? To be better on, I guess it just kind of makes you a burden sometimes. (YA5)*

## Discussion

To the author’s knowledge, this is among the first studies to qualitatively investigate the mental health experiences of young people living with T1D from a trauma-informed care and medical trauma perspective. Three themes were identified: (1) characterising potential traumatic events; (2) the long-term mental health impacts; and (3) the importance of resilience in coping and mental health recovery after a trauma experience. These findings complement existing understandings of mental health outcomes of young people living with T1D, particularly regarding the impact of stress on the young person, the role of the individual and their support systems, and the significance of developing diabetes resilience in young people.

### Potential sources of trauma

As depicted in the first and second themes, various potential sources of trauma were disclosed by the young people from pre-diagnosis through to ongoing management. These were accompanied by responses to adapt to the diagnosis and management of T1D but may have snowballed into more serious mental health outcomes. This journey is consistent with the current model of PMTS [17]. However, what is novel in our findings is that young people spoke of pre-diagnostic (i.e., symptom-onset) experiences as a potential source of trauma. These experiences include social adversity from family, teachers, and other support systems. This perceived adversity could be problematic to the trajectory of both psychological responses and attitudes towards T1D for the young person living with T1D. The PMTS model acknowledges that pre-existing psychological functioning of the young person and their families is vital to their psychological responses to the onset, diagnosis, and ongoing management of T1D [1]. Therefore, if young people perceive others’ negative reactions to the onset of diabetes symptoms as traumatic, this may worsen their own negative attitudes towards T1D, management, and even support systems post-diagnosis.

This finding has positive implications. Given the emergence of population-based strategies for early screening for T1D risk in the general population [18], there may be opportunities for models of care intervening in the pre-clinical stages of T1D to change the medical trauma trajectory by attenuating onset of diabetes symptoms alongside strategies that address pre-existing psychosocial and mental health concerns and higher risk adverse thought patterns that raise the risk of medical trauma later when T1D is confirmed. If the family of a young person identified to be highly likely to develop T1D was better equipped to handle the future demands of T1D, then it might be easier to manage the psychological responses in both the young people and their family members post-diagnosis. However, it will be important to also recognise the potential psychological impacts of screening itself, as screening for high risk of developing T1D can take a toll on young people and families alike [19].

The young people spoke of trauma at diagnosis, the severity of symptoms, and the necessary but invasive medical care to diagnose and treat their T1D. However, what was notable during the discussion of diagnosis and trauma was the perceived quality of clinical care received at the time of diagnosis, and how this seemed to influence the young people’s psychological responses post-diagnosis. Most of the literature investigating the experiences of young people and their families at the time of diagnosis does not explicitly discuss clinical procedures but infers a lack of trauma-informed care. For instance, a qualitative study by Robinson noted that at diagnosis, clinicians spoke of the consequences of unmanaged diabetes which contributed to a poorer outlook on young people’s diabetes and sense of self [20]. Understandably, existing nuanced exploration of diagnostic experiences may remain scant as diagnosis of T1D usually occurs at a young age, and symptoms may be so severe that the patient may not consciously remember the process.

The lack of trauma-informed care extends beyond the time of diagnosis. Young people in the current study noted that clinical processes elicited emotional responses that exacerbated poor outlook on T1D and sense of self. Namely, the invasive nature of clinical practice from diagnosis through to the adaptation process. The young person’s sociological circumstances, which includes their healthcare professionals, are pivotal to mental health recovery and ongoing willingness to engage with care [1]. Therefore, adverse experiences within the clinical care context may jeopardise the post-traumatic growth pathway and may enable avoidance of necessary diabetes-related management [21].

Considering that young people may perceive some aspects of clinical care as traumatic, developing robust guidelines regarding trauma-informed care for young people with T1D and additional training for clinicians may be beneficial to minimise further adverse experiences. A previous qualitative study reported that sensitive and person-centred communication results in open sharing of experiences and knowledge between the young person and their healthcare provider [22]. The core tenets of trauma-informed care pertain to choice, understanding, preventing re-traumatisation, and instilling hope [23]. If a young person already has a poor outlook on their sense of self or their diabetes due to potentially traumatic experiences, usual care (i.e., not trauma-informed care) may be perceived as traumatic and hinder mental health recovery and meaningful engagement in diabetes management.

Finally, young people noted some of the expected sources of trauma related to symptoms of T1D, albeit not to the degree that we expected. Previous literature noted that hyperglycaemia [9], hypoglycaemia, DKA, and recurring visits to the hospital as traumatic experiences but these were barely mentioned in a recent cohort of young people [7]. It could be the case that this sample of young people have not experienced these frequently, which may speak to how well they have managed their diabetes thus far.

### Normative versus maladaptive responses to T1D

The second theme generated in this study discusses the range of psychological responses to living with T1D, to which most responses may be considered normative to adapt to the demands of the illness. The young people described diabetes distress, resentment towards diabetes, grief, and a forced acceptance of having to live with the disease. These responses are especially common in young people who are living with T1D, especially as T1D is commonly described as relentless [24]. Everyday processes and activities, especially for young people, are often overshadowed by demands of T1D. For instance, eating, sleeping, and playing cannot be optimally performed without considering the impact on blood glucose levels [25] or an insulin plan. These extra layers of cognitive burden required to manage T1D inevitably impact a young person at some point in their journey. Given these circumstances, a greater proportion of the broad range of psychological responses may be considered normative for this population.

Viewing T1D through the different stages of development during adolescence and young adulthood also makes these ‘normative’ responses more poignant. This transition is often a stressful time for adolescents and young adults (e.g., transition to high school, further study, working) [26]. The pressures of being an adolescent or a young adult is compounded by pressures faced by young people managing their T1D. The interplay between diabetes and common stressors among young people are well-documented, such that stressors in daily life (accounting for diabetes and non-diabetes related concerns) may ultimately impact diabetes management. This can result in poor diabetes-related outcomes (i.e., affecting BGL and HbA1c management) and impact day to day activities as well [24].

What we can consider, however, is whether these normative responses may eventually develop into more severe, maladaptive responses. Young people in the present study have described instances where relationships with others, themselves, and T1D have been negatively impacted by the psychological sequalae of the unresolved trauma. Ultimately, this negatively affects engagement to care as avoidance from engaging with support systems including family members and their clinical care team are common experiences. Further examples of the more severe responses in our study have been suicidality, self-harm behaviours, and self-stigma. Hopelessness and perceived burdensomeness are well-understood to be determinants to suicidality and self-harm behaviours in the general population [27]. It is unsurprising that these extreme maladaptive responses were reported, granted that the determinants (e.g., hopelessness and perceived burdensomeness) were present and accounted for as normal responses.

### Resilience and post-traumatic growth

The conceptualisation of resilience by young people throughout the diabetes journey is also congruent with the existing framework of diabetes resilience [28]. The current iteration of the framework recognises the interplay between the individual and interpersonal support systems with regards to protective psychological processing (i.e., benefit-finding, hopefulness, optimism), and how this might impact diabetes competence (referred to as behavioural resilience). These psychological processes influence the level of competence required for diabetes management while minimising the impact of stress. Diabetes resilience is also acknowledged to be an act of engaging in management, such that it can be practice rather than a passive state of being. The current model also describes the impact of general resilience which describes key developmental milestones such as social and academic pressures which are present throughout adolescence and young adulthood.

The diabetes resilience framework does not wholly describe the points along the journey where resilience plays a role, when it develops, and how it develops [28]. However, it does acknowledge that resilience is dynamic. The findings of the present study can assist in explaining this relationship. General resilience developed by young people prior to diagnosis, particularly from social and familial support, was impactful when adapting to the demands of diabetes management in the early stages post-diagnosis. In the case of our sample, this impacted the young people’s outlook and willingness to engage in diabetes management and establishment of parents as crucial support systems throughout the journey. For instance, we can juxtapose the young people whose journey started with strong familial support versus those who did not (thus impacting outlook on T1D and its management), and how well they are currently handling diabetes management; their mental health outcomes, relationship with diabetes, and outlook on life are vastly different. We posit that perhaps the difference between the two groups is how well-established general resilience was leading up to diagnosis.

We understand that resilience, especially diabetes resilience, is predominantly a practice, not a state of being. Therefore, diabetes resilience may be practiced and built immediately post-diagnosis and beyond. Congruent with the diabetes resilience framework, our participants discussed that engaging in protective factors (i.e., integration to community, supportive family, and developing competence to managing T1D on their own) indeed were noted to improve relationship with T1D. While diabetes resilience is being built, perhaps it is general resilience which was practiced prior to diabetes onset that prevents the young people from succumbing to the pressures of diabetes management in the early stages post-diagnosis at the individual level. Once diabetes resilience is sufficiently practiced, which might vary depending on the circumstances of the young person, then the interplay between diabetes resilience and general resilience can be established.

Our findings highlight the importance of support systems such as family and friends among our sample of young people and are consistent with both the diabetes resilience and PMTS frameworks. It is commonly discussed in the literature that parents and peers are important in sharing the burden of diabetes management [29, 30], although potentially to the detriment of the supporter’s own wellbeing [31]. However, control and the transfer of T1D management can be difficult during the transition to adulthood [32]. Excessive control by parents might be detrimental to the development of diabetes resilience, as it can lead to resistance from the young person. Some of the young people in the present study discuss this relationship dynamic, such that some viewed that their parents navigated the concept of control well, while others felt that their parents were overbearing which impacted their willingness to engage with their diabetes management.

### Strengths and limitations

The present study is subject to some limitations. Firstly, participation in the study required that young people were at least 13 years of age. By this time, a participant would already be settling into high school. As such, an exploration of the transition from pre-adolescence to adolescence was not possible. This transition is typically associated with a significant shift in self-perception, social, and academic circumstances which would pose their own unique concerns. Secondly, only a handful were diagnosed when they were in their childhood years. Because of this, exploration of how diabetes management, mental health, and diabetes outcomes evolved from childhood through to adolescence and young adulthood might be lacking nuance so this warrants further investigation. Finally, recall bias may be present particularly among those who are further away from their age of diagnosis, especially if adverse events were close to the age of diagnosis. The recall bias might be compounded by potential maladaptive coping from adverse events and thus might influence how events, memories, and feelings are discussed at the time of the interview.

However, the present study also has its strengths. Since the participants ranged from the early stages of adolescence through to young adulthood (some young adults surpassing their mid-twenties), we were able to find distinctions between the adolescence, young adulthood, and the transition between the two which may influence mental health and diabetes outcomes with references to the PMTS model. The range of responses from the participants and their experiences with their families is an asset to our investigation, such that we can hypothesise the effects of the presence, or lack of, certain constructs (e.g., the development of diabetes resilience).

Finally, and arguably the most important strength of the study, this is among the first to qualitatively explore the experiences of young people with T1D through the lens of medical trauma and trauma-informed care. Previous studies investigating trauma in this population have attempted to quantitatively ascertain the prevalence and sources of trauma, although this area of research requires further exploration. The experience of medical trauma is not routinely collected in clinic which highlights the novelty of the findings of the study.

## Conclusion and implications

The present study has provided insight into medical trauma and the mental health experiences of young people living with T1D. The young people in the present study stated that they have experienced medical trauma, described the sources of their medical trauma, explained their coping strategies in response to this trauma, and ultimately have described the importance of resilience to overcome the challenges of living with T1D. These findings are novel. Given the nuanced description of the experiences of young people in the study, we have utilised these findings to co-design a trauma-informed, psychosocial intervention with young people living with T1D, their parents, and their treating clinicians to equip young people with skills to develop their diabetes resilience and reduce the impact of stress. We recommend that clinics develop and implement measures routinely to consider medical trauma sensitive models of care, and screen for medical trauma in young people with T1D, and its potential impact on other mental health outcomes, diabetes management, and outcomes.

## Supplementary Information

Below is the link to the electronic supplementary material.

Supplementary Material 1

## Data Availability

The raw data produced in this study cannot be shared due to ethical reasons pertaining to participant privacy and identifiability.

## Footnotes

1 Note that the inclusion of the mention of race in this scenario is to showcase the emotional response to a stimulus (e.g., aversion), rather than introducing a new construct to the themes pertaining to the clinician’s actual race.
