# Supplementary material for: Application of the paediatric medical traumatic stress model to the mental health experience of young people living with type 1 diabetes: a qualitative study
Source: BMC Psychiatry. 2026 Jan 29;26:125. doi: 10.1186/s12888-025-07760-w (PMC12879457; doi:10.1186/s12888-025-07760-w)
Supplement: Supplementary file 1 — Supplementary Material 1 [file 12888_2025_7760_MOESM1_ESM.docx]

**Interview guide**

| *A community-led, trauma-informed psychosocial intervention to improve health outcomes of children and young people with Type 1 Diabetes: Wellbeing-T1D* |
| --- |

1. **Introduction**

- Introduce facilitator (name, professional background, role in the project).
- Thank you for agreeing to share your experiences about being diagnosed and living with Type 1 Diabetes (T1D).
- Being diagnosed with T1D and ongoing management of T1D can be quite stressful.
- As we know, going to hospital a lot and being sick is very common for young people with type 1 diabetes, and this can result potentially in mental health problems that make it even more difficult to manage diabetes daily.
- This stress might look like some young people may not managing their diabetes and some people may even avoid going to their clinic appointments which does not bode well in terms of diabetes management.
- The aim of these sessions is to co-design a mental health program with community members (you all here), researchers and clinic experts working in the T1D space. This program aims to help young people boost their resilience and improve their wellbeing to prevent or recover from the stress associated with T1D.
- The purpose of these interviews is to find out how the program should be delivered and what should be included.
- Today’s discussion will involve exploring mental health experiences, planning, exploring, and developing ideas about the program.

1. **Reminders for today**

- Before we start, I would like to remind you that all the information you provide is treated as strictly confidential and will not be released by investigators unless required to do so by law (such as harming one’s self or others, or discussing illegal activities). This means that your name, or any information that can identify you, will not be used in any reports or other aspects of the research.
- If you inadvertently disclose any notifiable illegal activities during today’s interview, then I may have to complete a statuary declaration/disclose the information that I have heard.
- Your participation is completely voluntary. You don’t have to answer any questions that you don’t want to, and you can stop at any time.
- We will be recording our conversation today so that we can accurately recall everything we’ve spoken about. Do we still have your permission to record?
- Do you have any questions before we get started?

1. **Icebreaker**

- Invite participants to introduce themselves and share one activity/tool/resource that they use to help them relax. Facilitators can go first to set an example (e.g., “I’m Alix, I’m a big fan of mindfulness apps to help me meditate – like the smiling mind app”).

1. **Key questions for exploration of mental health experiences**
2. Can you tell me a bit about how you are going?
3. How are you finding living with Type 1 Diabetes?
4. Is there anything about Type 1 Diabetes that you find challenging?
5. What are the main feelings you usually feel when you think about diabetes?
   1. What do you do when you have these feelings?
   2. Do you reach out to people when you feel hard emotions? If so, who?
   3. Have you engaged with mental health professionals before?
6. What has helped you get through these tough feelings when managing your diabetes?
7. Are there other things in your life that make you feel better or worse about your diabetes?
8. **Conclusion**

- Thank you for spending the time to join us today.
- Check how participant/s are feeling after the interview. Remind participant/s of the emergency and service contact info if they need it and encourage a self-care task following the interview.
